# Hyperdiluted Triamcinolone Injections for Nasal and Alar Contouring

**DOI:** 10.1111/jocd.70861

**Published:** 2026-04-25

**Authors:** Kyu‐Ho Yi, Suyeon Lee, Jong Keun Song, Jin‐Hyun Kim, Arash Jalali, Dagne Pupo

**Affiliations:** ^1^ You and I Clinic Seoul Republic of Korea; ^2^ Medical Research Inc. Wonju Republic of Korea; ^3^ Pixelab Plastic Surgery Clinic Seoul Republic of Korea; ^4^ One Clinic Vancouver Canada; ^5^ Dagné Pupo Clinic Mallorca Spain

**Keywords:** cosmetic techniques, glucocorticoids, injections, Intralesional, nose, triamcinolone Acetonide

## Abstract

**Background:**

Demand for minimally invasive nasal contour refinement has increased, particularly among patients seeking subtle esthetic improvement without surgery or volume augmentation. While hyaluronic acid filler‐based nonsurgical rhinoplasty can alter contour, it fundamentally adds volume and carries rare but severe vascular risks. In contrast, intralesional corticosteroids are known to induce localized soft‐tissue thinning and have been used in postoperative rhinoplasty to manage supratip fullness, suggesting a potential role in nonsurgical soft‐tissue contour modulation.

**Objective:**

To describe a nonsurgical, nonfiller technique using hyperdiluted triamcinolone acetonide (TAC) microinjections for targeted reduction of nasal and alar soft‐tissue fullness, and to present a structured case‐series protocol with standardized Global Aesthetic Improvement Scale (GAIS) reporting.

**Results:**

Twenty adult patients underwent a standardized 10‐point microinjection protocol using hyperdiluted TAC (approximately 1.3 mg/mL), with 1.0 mL administered per session and sessions repeated every 3 weeks for a total of five sessions. The cumulative TAC dose per patient was approximately 6.5 mg. Outcomes were assessed using patient‐reported GAIS at final follow‐up. This manuscript provides a patient‐level GAIS reporting framework and an illustrative results table to support transparent outcome documentation. The technique is conceptually distinct from surgical rhinoplasty and filler‐based approaches, aiming for gradual, localized soft‐tissue thinning through dose‐minimized, distributed corticosteroid exposure.

## 1 Introduction

The modern esthetic patient increasingly seeks nasal refinement with reduced downtime, lower cost, and avoidance of irreversible structural change. Surgical rhinoplasty remains the gold standard for major shape correction and functional goals, but it requires anesthesia, recovery time, and carries the inherent unpredictability of wound healing and scar dynamics. Even when technically successful, soft‐tissue behavior—particularly in thick‐skinned noses—may obscure definition for months and contribute to supratip fullness or persistent bulk. In parallel, “nonsurgical rhinoplasty” has gained popularity, most commonly using hyaluronic acid (HA) fillers to camouflage dorsal irregularities, improve perceived projection, or smooth contour transitions.

However, filler‐based nasal reshaping is fundamentally an augmentation strategy: it adds volume to change light reflection and apparent proportions. While effective for select indications, it cannot reduce tissue volume. Additionally, the nose is considered a high‐risk region for vascular compromise because of its arterial connections and the potential for embolic or compressive vascular occlusion. Contemporary literature confirms that most complications of nonsurgical rhinoplasty are mild, yet severe vascular events—including skin necrosis, vision loss, and stroke—have been reported and remain a central safety concern [1, 2, 3, 4]. Case reports document skin necrosis after HA filler nasal injections despite rescue attempts, emphasizing that “rare” does not mean “impossible” and that consequences may be permanent or disfiguring [3]. Systematic reviews similarly highlight clinical effectiveness while underscoring the reality of serious vascular complications within the published literature [1]. These issues create a clinical gap for patients whose primary esthetic concern is soft‐tissue fullness—for example, a thick soft‐tissue envelope along the nasal sidewall, supratip region, or alar lobule—where adding filler may worsen bulk and surgery may be more invasive than desired. This gap is particularly relevant when the esthetic objective is subtle contour refinement rather than skeletal restructuring. Intralesional corticosteroids, particularly triamcinolone acetonide (TAC), have a long history of use in dermatology and facial plastic surgery for modulation of fibrosis, scar behavior, and soft‐tissue thickness. Standard intralesional TAC is widely used for hypertrophic scars and keloids, typically at concentrations of 10–40 mg/mL, with lower concentrations often selected for facial sites to reduce adverse effects [5, 6]. Importantly, the known and expected local effect of intralesional TAC includes dermal and subcutaneous thinning and lipoatrophy—outcomes that are usually categorized as adverse events but may be reframed as controlled therapeutic endpoints when the clinical goal is soft‐tissue reduction [7, 8].

Within rhinoplasty practice, TAC injections have been described for the management of postoperative supratip fullness (“soft‐tissue pollybeak”), often as a first‐line measure before revision surgery. Hanasono et al. reported intralesional TAC as an effective nonsurgical treatment for soft‐tissue pollybeak deformity caused by subdermal scarring [9]. Randomized and prospective studies further suggest that postoperative TAC can reduce edema and demonstrate measurable thinning of the nasal skin envelope, supporting the biological plausibility of steroid‐mediated soft‐tissue reduction [10, 11]. A recent scoping review situates steroid injection among established nonsurgical strategies for postoperative soft‐tissue pollybeak, while also emphasizing the need for standardized and objective outcome measures in future trials [12].

The present technique is neither a rhinoplasty nor a filler‐based “liquid nose job.” Rather than adding volume or surgically altering cartilage and bone, it attempts to gently atrophy targeted soft tissue using hyperdiluted TAC delivered as multiple microinjections in a symmetric pattern. This concept also relies on TAC's formulation characteristics: TAC suspensions have relatively low solubility compared with more water‐soluble corticosteroids, supporting a depot effect and prolonged local activity at the injection site [13, 14]. A crucial design feature of this protocol is dose minimization. TAC 40 mg/mL is diluted into a large volume of normal saline (30cc), yielding a working concentration of approximately 1.3 mg/mL. With 1.0 mL injected per session, the total TAC delivered per session is approximately 1.3 mg, distributed across 10 injection points (~0.13 mg per point). Over five sessions, cumulative TAC exposure is approximately 6.5 mg. This dose is small relative to systemic dosing ranges described in product labeling, where adrenal suppression has been reported following intramuscular doses of 60–100 mg [13]. The intent of hyperdilution and multipoint distribution is to reduce the risk of excessive focal atrophy while permitting gradual, localized contour change through repeated, low‐dose exposure.

Given the limited literature on nonpostoperative, esthetic nasal soft‐tissue reduction using hyperdiluted TAC microinjections, a structured case‐series report paired with standardized outcome documentation is a practical first step. The Global Aesthetic Improvement Scale (GAIS) is a widely used, simple five‐point scale comparing post‐treatment appearance with baseline and has been frequently applied in esthetic injectable studies [15]. In the sections below, we present a detailed protocol and a patient‐level GAIS reporting framework to support transparent documentation of outcomes.

## 2 Materials and Methods

### 2.1 Study Design and Participants

This study was designed as a prospective, single‐arm case series involving 20 adult patients seeking nonsurgical nasal and alar contour refinement through targeted soft‐tissue reduction. All participants provided written informed consent for treatment and standardized clinical photography.

Inclusion criteria for manuscript completeness were as follows: adults aged 18 years or older requesting nasal or alar contour refinement without a desire for surgical intervention; a predominant esthetic concern related to soft‐tissue fullness of the nasal sidewall, supratip region, or alar lobule rather than major structural deformity; and willingness to complete five treatment sessions with follow‐up evaluation. Exclusion criteria included pregnancy or breastfeeding; active local infection or inflammatory dermatosis at planned injection sites; a history of poor wound healing, uncontrolled diabetes, or immunosuppression; prior nasal filler injection within a defined washout period (e.g., 12 months); and known hypersensitivity to TAC or its excipients.

### 2.2 Injection Formulation and Dosing

**FIGURE 1:**
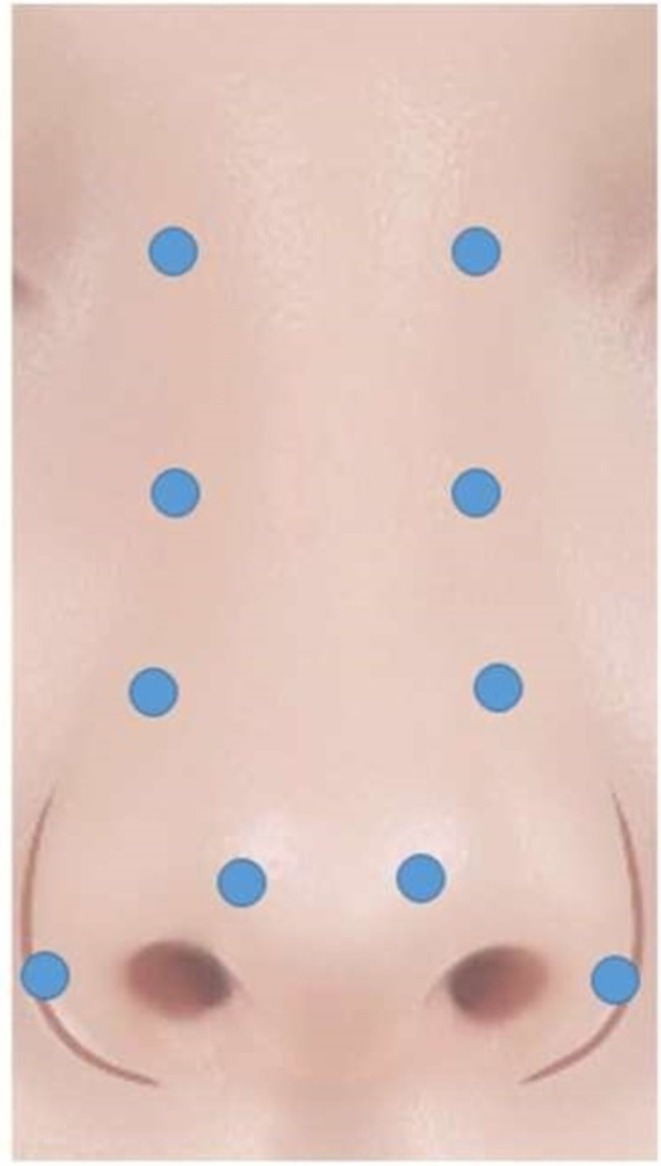
Standardized injection protocol for hyperdiluted triamcinolone acetonide microinjection. Schematic illustration of the standardized bilateral 10‐point injection pattern used for hyperdiluted triamcinolone acetonide microinjection. Injection points are symmetrically distributed along the upper, mid, and lower nasal sidewalls and dorsum, as well as the supratip and lower lateral alar regions. Each point received 0.1 mL of diluted triamcinolone acetonide, resulting in a total injection volume of 1.0 mL per treatment session.

A suspension of TAC 40 mg/mL (1 mL) was mixed with 30 mL of normal saline, producing approximately 31 mL of solution with a final TAC concentration of approximately 1.3 mg/mL. This hyperdilution strategy was selected to minimize focal corticosteroid exposure while allowing distributed, low‐dose delivery across multiple injection points. Per treatment session, a total volume of 1.0 mL was administered. Injections were delivered across 10 predefined points, with 0.1 mL injected at each point. The approximate TAC dose per session was therefore 1.3 mg, corresponding to approximately 0.13 mg per injection point. Treatment sessions were repeated at 3‐week intervals for a total of five sessions, resulting in a cumulative TAC exposure of approximately 6.5 mg per patient. This cumulative dose is substantially lower than systemic corticosteroid dosing ranges described in product labeling, where adrenal suppression has been reported following intramuscular doses of 60–100 mg [13]. A standardized bilateral 10‐point injection pattern was used for all patients, as illustrated in Figure 1.

### 2.3 Injection Pattern

Injection sites were distributed symmetrically over the upper, mid, and lower nasal sidewalls and dorsum, the supratip or tip‐adjacent soft tissue, and the lower lateral alar region. This pattern was designed to achieve even distribution of the hyperdiluted corticosteroid while avoiding excessive focal deposition. The injection map and session structure are summarized in Table 1. The general concept of intralesional steroid use for nasal soft‐tissue modulation is supported by prior rhinoplasty literature describing steroid injections for postoperative supratip fullness [9, 10, 11, 12].

**TABLE 1:** Standardized 10‐point injection protocol for hyperdiluted triamcinolone acetonide.

Injection Region	Number of Points	Volume per Point (mL)	Total Volume (mL)
Upper nasal sidewall/dorsum	2	0.1	0.2
Mid nasal sidewall/dorsum	2	0.1	0.2
Lower nasal sidewall	2	0.1	0.2
Supratip/tip‐adjacent area	2	0.1	0.2
Alar region	2	0.1	0.2

### 2.4 Outcome Measures

The primary outcome measure was patient‐reported GAIS assessed at the final follow‐up visit, 3 weeks after the fifth treatment session. GAIS is a five‐point ordinal scale comparing post‐treatment appearance with the pretreatment baseline and is widely used in esthetic medicine for injectable treatments [15]. The scale ranges from “very much improved” to “worse,” with “no change” representing no difference from baseline. Standardized clinical photographs were obtained at baseline and at each follow‐up visit using frontal, lateral, and oblique views under consistent lighting and positioning conditions.

### 2.5 Safety Monitoring

Patients were evaluated at each visit for local adverse events, including bruising, erythema, pain, hypopigmentation, telangiectasia, and signs of excessive skin thinning or soft‐tissue atrophy. Patients were also queried regarding systemic symptoms potentially associated with corticosteroid exposure. Known local adverse effects of intralesional corticosteroid injections include soft‐tissue atrophy and pigmentary alteration, which were specifically monitored throughout the treatment course [7, 8]. No formal laboratory monitoring was performed given the low cumulative corticosteroid dose used in this protocol.

### 2.6 Statistical Analysis

Given the exploratory nature of this case series, statistical analysis was descriptive. GAIS outcomes were summarized using frequencies and proportions. Improvement was defined as a GAIS score of 1–3 at final follow‐up. Formal hypothesis testing was not performed, as GAIS is inherently comparative to baseline and the study was not designed for inferential statistical analysis.

## 3 Results

### 3.1 Notes on GAIS “before vs. after” Assessment

The GAIS is defined as a comparative assessment relative to baseline appearance rather than an independent baseline score. In many esthetic studies, GAIS is recorded only after treatment, with baseline serving as the reference state [15]. However, because this manuscript aims to present patient‐level “before and after” reporting, baseline status was anchored as GAIS score 4 (“no change”) by definition. This approach allows tabular presentation of pre‐ and post‐treatment GAIS values while preserving the comparative meaning of the scale (Table 2).

**TABLE 2:** Global Aesthetic Improvement Scale (GAIS).

Score	Description
1	Very much improved
2	Much improved
3	Improved
4	No change
5	Worse

### 3.2 Patient‐Reported GAIS Outcomes

**FIGURE 2:**
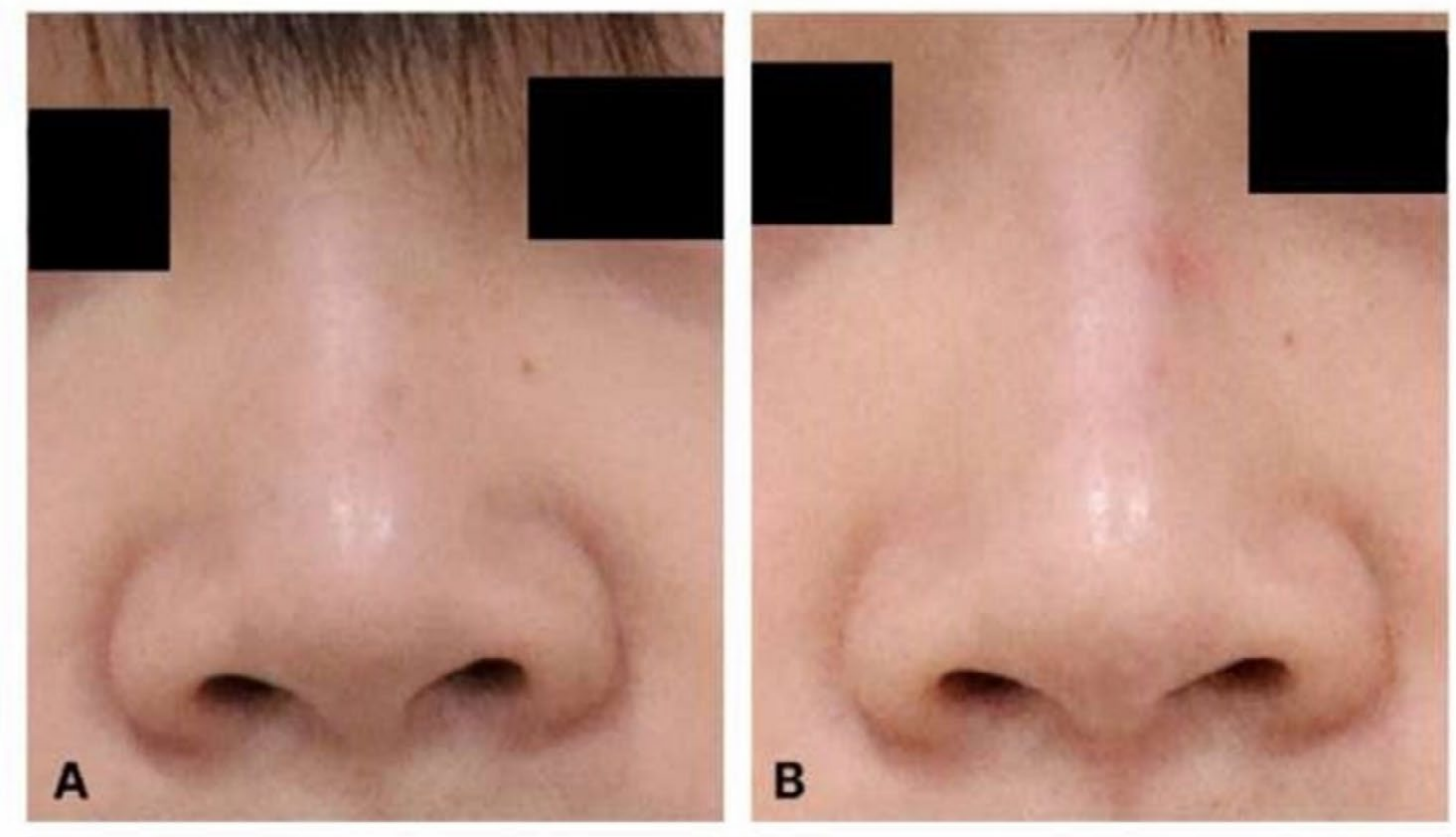
Representative clinical case: Patient 1. Representative before‐and‐after clinical photographs of Patient 1 undergoing hyperdiluted triamcinolone acetonide microinjection for nasal and alar soft‐tissue contour refinement. (A) Frontal view before treatment. (B) Frontal view at final follow‐up after completion of five treatment sessions.

**FIGURE 3:**
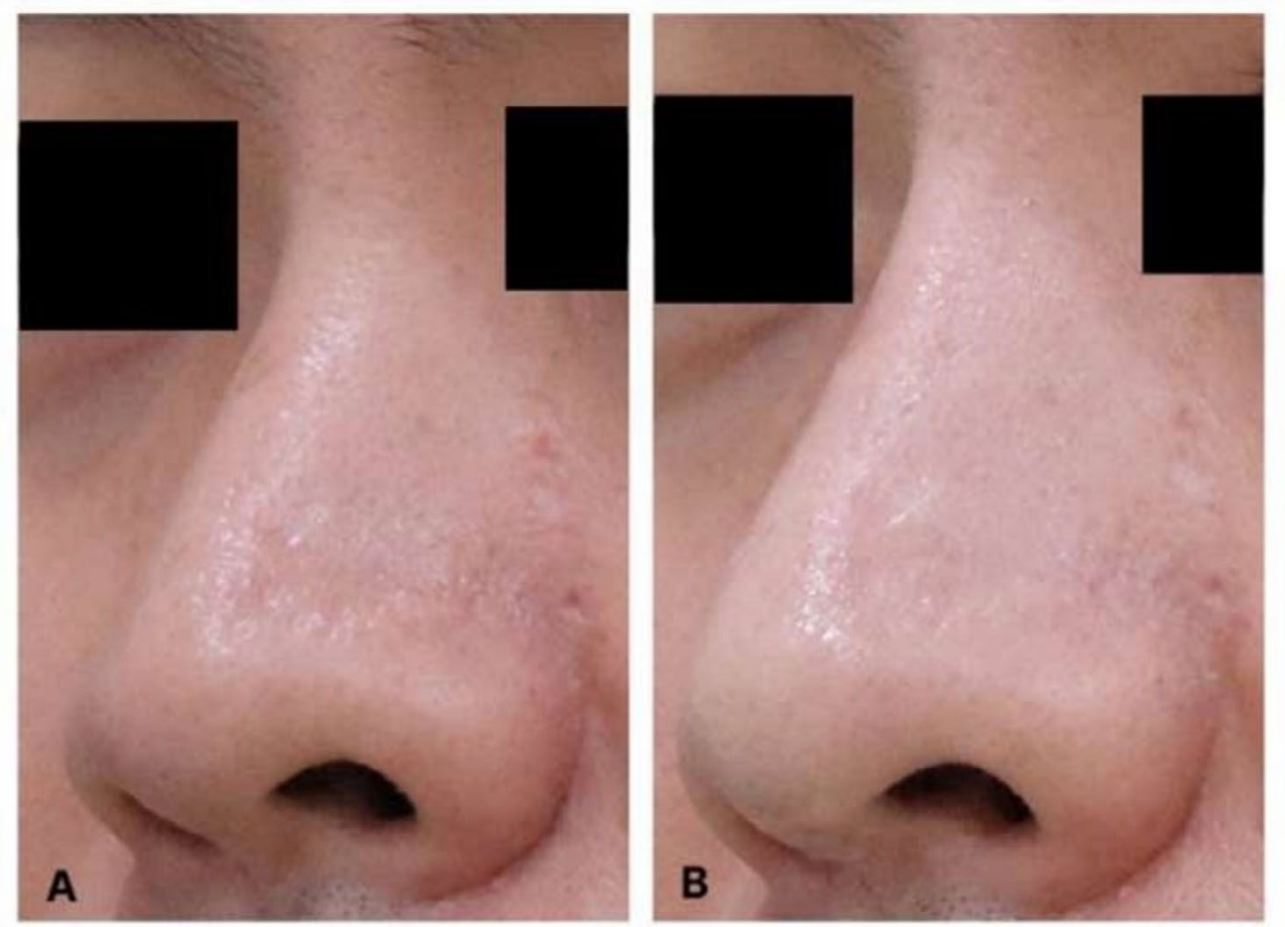
Representative clinical case: Patient 3. Representative before‐and‐after clinical photographs of Patient 3 undergoing hyperdiluted triamcinolone acetonide microinjection for nasal and alar soft‐tissue contour refinement. (A) Oblique view before treatment. (B) Oblique view at final follow‐up after completion of five treatment sessions.

Representative clinical photographs demonstrating the application of this protocol in Patient 1 are shown in Figure 2. An additional representative case (Patient 3) is presented in Figure 3 to illustrate treatment outcomes in an oblique view. Patient‐reported GAIS outcomes at the final follow‐up visit (3 weeks after the fifth treatment session) are summarized descriptively using a patient‐level reporting framework (Table 3). GAIS scores ranged from “very much improved” to “no change,” reflecting variable degrees of perceived esthetic improvement following treatment. The results are presented to illustrate the distribution of patient‐reported outcomes within the treated cohort.

**TABLE 3:** Patient‐reported GAIS outcomes at final follow‐up.

Patient	GAIS Score
Patient 1	Improved
Patient 2	Improved
Patient 3	Improved
Patient 4	Improved
Patient 5	Improved
Patient 6	Improved
Patient 7	Improved
Patient 8	Improved
Patient 9	Improved
Patient 10	Improved
Patient 11	Improved
Patient 12	Improved
Patient 13	Improved
Patient 14	Improved
Patient 15	Improved
Patient 16	Improved
Patient 17	Improved
Patient 18	Improved
Patient 19	Improved
Patient 20	Improved

## 4 Discussion

This case series framework describes a minimally invasive strategy for nasal and alar contour refinement that is deliberately positioned as neither surgery nor filler‐based nonsurgical rhinoplasty. This distinction is not merely semantic; it reflects differences in esthetic goals, mechanisms of action, durability expectations, complication profiles, and counseling considerations. Representative clinical cases illustrating this approach are shown in Figures 2 and 3.

### 4.1 Conceptual Difference From Surgery

Surgical rhinoplasty alters the nasal framework through modification of bone and cartilage and their supporting structures. Even when the esthetic goal is limited to improved definition, the method remains structural and invasive, involving resection, reshaping, grafting, or repositioning. Postoperative healing introduces variability related to edema, fibrosis, and scar maturation, and thick soft‐tissue envelopes may obscure refinement for prolonged periods. Revision procedures are not uncommon, and recovery time, cost, and surgical risk must be considered.

In contrast, the hyperdiluted TAC technique described here does not attempt to reshape the skeletal framework. Instead, it targets the soft‐tissue envelope—specifically perceived fullness along the nasal sidewalls, supratip region, and alar lobule. This conceptual positioning aligns with rhinoplasty literature in which intralesional TAC has been used to manage postoperative supratip fullness (“soft‐tissue pollybeak”) before considering revision surgery [9]. Additional studies have demonstrated that postoperative TAC injections can reduce edema and contribute to measurable thinning of the nasal skin envelope, supporting the biological plausibility of steroid‐mediated soft‐tissue modulation [10, 11]. A recent scoping review further situates steroid injection among established nonsurgical strategies for postoperative soft‐tissue pollybeak, while emphasizing the need for standardized outcome measures [12].

### 4.2 Conceptual Difference From Filler‐Based Approaches

HA filler‐based nonsurgical rhinoplasty has gained popularity because it can produce immediate contour change without surgical downtime. However, filler rhinoplasty functions fundamentally by adding volume. While this is advantageous for indications such as a low radix or dorsal irregularities, it may be counterproductive in patients whose primary concern is soft‐tissue thickness or bulk, particularly in the alar or sidewall regions. Safety considerations also differ substantially. Although most filler rhinoplasty procedures are uncomplicated, severe vascular complications—including skin necrosis, visual loss, and stroke—have been repeatedly documented in the literature [1, 2, 3, 4]. Systematic reviews and case reports emphasize that these events, while rare, can be catastrophic and permanent [1, 3]. Meta‐analytic and review literature underscores that prevention depends on meticulous technique and detailed anatomical knowledge but cannot entirely eliminate risk [2, 4]. The existence of these risks does not negate the utility of fillers; rather, it highlights the potential value of alternative approaches for patients seeking contour refinement without volume augmentation and who may be risk‐averse to intravascular filler complications.

The hyperdiluted TAC approach shifts the dominant risk profile away from filler‐related vascular events toward steroid‐specific local tissue effects. As such, it does not represent a “safer” version of filler rhinoplasty, but rather a conceptually distinct intervention with different mechanisms, benefits, and risks.

### 4.3 Rationale for TAC and Hyperdilution

TAC is one of the most commonly used intralesional corticosteroids and has a long history of application in dermatology and facial plastic surgery. Its local effects include modulation of inflammation, inhibition of fibroblast activity, reduced collagen synthesis, and potential adipocyte atrophy. These effects are typically considered adverse in many clinical contexts but may be therapeutically useful when controlled soft‐tissue reduction is the desired outcome [7, 8]. An important pharmacologic feature of TAC is its relatively low solubility compared with more water‐soluble corticosteroids, resulting in a suspension formulation with prolonged local activity and a depot‐like effect at the injection site [13, 14]. This characteristic supports its selection for applications where sustained local tissue interaction is desired. Hyperdilution was employed in this protocol for two primary reasons. First, dose minimization: standard intralesional TAC concentrations for hypertrophic scars and keloids commonly range from 10 to 40 mg/mL, with lower concentrations often recommended for facial use to reduce adverse effects [5, 6]. In contrast, the working concentration used here was approximately 1.3 mg/mL, with a total of approximately 1.3 mg administered per session. Over five sessions, cumulative exposure was approximately 6.5 mg—substantially lower than systemic dosing levels associated with adrenal suppression, which have been reported following intramuscular doses of 60–100 mg [13]. Second, spatial distribution: by dividing the total volume across 10 injection points, each site received a very small dose, aiming to reduce the risk of excessive focal atrophy while allowing gradual, diffuse contour change.

### 4.4 Interpretation of GAIS Outcomes

The GAIS was selected as the primary outcome measure because of its simplicity and widespread use in esthetic injectable studies [15]. GAIS provides a global, patient‐centered assessment of esthetic change relative to baseline and is practical for early case series. However, GAIS has inherent limitations. It is subjective, susceptible to expectation bias, and does not localize improvement to specific nasal subunits. For this reason, the present manuscript emphasizes transparent, patient‐level reporting rather than inferential statistical claims. Future studies would benefit from pairing GAIS with objective or semiobjective measures, such as blinded evaluator assessments, standardized anthropometric ratios, or three‐dimensional imaging, as has been advocated in broader rhinoplasty and esthetic outcomes literature [12].

### 4.5 Safety Considerations and Patient Selection

Hyperdiluted TAC microinjection is not without risk. Local adverse effects of intralesional corticosteroids include skin and subcutaneous atrophy, hypopigmentation, and telangiectasia [7, 8]. In the present protocol, these risks are addressed through conservative dosing, hyperdilution, and multipoint distribution, but they cannot be eliminated. Careful patient selection, precise injection technique, and thorough informed consent are therefore essential. Systemic corticosteroid effects are theoretically possible but are expected to be unlikely given the low cumulative dose used in this protocol [13]. Nonetheless, standard screening for contraindications to corticosteroid use remains appropriate.

### 4.6 Limitations and Future Directions

Several limitations should be acknowledged. This report represents a small, single‐arm case series without a control group. Outcomes are based on patient‐reported GAIS rather than objective measurements, and follow‐up duration may be insufficient to capture delayed steroid‐related tissue changes or long‐term durability. Additionally, generalizability may be limited by patient selection and injector technique.

Future investigations could include controlled comparisons with observation, standard‐dose focal TAC injection, or filler‐based approaches in carefully selected indications. Longer follow‐up and incorporation of objective outcome measures would further clarify safety, durability, and appropriate clinical indications.

## 5 Conclusion

This case series suggests hyperdiluted TAC microinjection for nasal and alar contour refinement represents a distinct conceptual category between surgery and filler‐based nonsurgical rhinoplasty. Rather than altering structure or adding volume, it aims to achieve gradual, localized soft‐tissue reduction through dose‐minimized, distributed corticosteroid exposure. Supported by established rhinoplasty literature on postoperative steroid use and by the known tissue effects of intralesional TAC [7, 8, 9, 10, 11, 12, 13, 14], this approach may offer an alternative option for selected patients seeking subtle contour refinement. Careful documentation, conservative technique, and further study are required to define its role within esthetic practice [16, 17].

## Author Contributions

Conceptualization: Kyu‐Ho Yi. Methodology: Kyu‐Ho Yi. Investigation: Kyu‐Ho Yi. Data curation: Kyu‐Ho Yi. Writing – original draft: Kyu‐Ho Yi. Writing – review and editing: Suyeon Lee, Jong Keun Song, Jin‐Hyun Kim, Arash Jalali, and Dagne Pupo. Supervision: Kyu‐Ho Yi. All authors reviewed and approved the final manuscript.

## Funding

The authors have nothing to report.

## Ethics Statement

This report was prepared in accordance with the principles of the Declaration of Helsinki. As revised, the manuscript is submitted as a technical note with representative cases from routine clinical practice rather than as a completed prospective clinical trial.

## Consent

Written informed consent was obtained from all patients for the publication of anonymized clinical images and data included in this manuscript.

## Conflicts of Interest

The authors declare no conflicts of interest.

## Data Availability

Data sharing not applicable to this article as no datasets were generated or analysed during the current study.
